# Seeing Is Ordering: The Influence of Mukbang Video Types on Consumers’ Immediate Ordering Intention

**DOI:** 10.3390/foods15091607

**Published:** 2026-05-06

**Authors:** Hui Tian, Xiaojing Qin, Defang Sha, Mengyao Li

**Affiliations:** 1 Zhujiang College, South China of Agricultural University, Guangzhou 510900, China; tianhui@scauzj.edu.cn (H.T.); shadefang@scauzj.edu.cn (D.S.); 2 Shanghai Cancer Institute, Shanghai 200032, China

**Keywords:** mukbang short videos, visual hunger, oral sensory arousal, immediate ordering intention, visual composition

## Abstract

With the rapid growth of short-video platforms and online food-delivery services, mukbang short videos have become an important influence on consumers’ immediate food-related decisions. Drawing on visual hunger theory, this research examines how different types of mukbang videos influence consumers’ immediate ordering intention, as well as the roles of oral sensory arousal and visual composition in this process. Across three preregistered studies (*N* = 713), the findings show that immersive mukbang increases consumers’ immediate ordering intention more than noisy mukbang. This effect occurs because immersive mukbang evokes stronger oral sensory arousal, and it becomes more pronounced when the video adopts a food-centered rather than human-centered visual composition. These findings extend visual hunger theory to the short-video food context and offer practical implications for the design of digital food content.

## 1. Introduction

With the growing popularity of short-video platforms and online food-delivery apps, food-related video content has become increasingly embedded in consumers’ momentary food decisions [1,2,3]. Among this type of content, mukbang videos have become one of the most unique types of digital food media [4,5]. Unlike conventional food advertising, mukbang videos do not only show food items, as ordinary food commercials do [6], but produce vivid consumption experiences using soundbites of chewing, swallowing signals, extreme focus on foods, and systematic patterns of consumption [7,8]. This means that mukbang could influence appetitive behaviors, as well as prompt consumer ordering behavior during actual consumption conditions [9,10].

Previous studies indicate that digital signals of food can have a significant effect on the appetite of consumers, their assessments, and behavioral patterns [11,12,13]. The visual hunger theory states that people are extremely vulnerable to food-related visual cues, and just the sight of food images or videos can evoke hunger, reward expectation, and preparedness to eat, regardless of any actual intake [13]. This is supported by neuroimaging studies showing that visual food cues are always associated with activation of parts of the brain involved in reward processing, motivation, and allocation of attention, suggesting that seeing food could translate into later tendencies of consumption and responses to choices [14]. Furthermore, food cue reactivity studies have shown that salient food stimuli cause increased craving and appetite and increased probability of food-related behavior [6,15].

Based on this foundation, studies of digital food video indicate even higher levels of perceptual involvement in response to movement in food images as opposed to images of food that are not moving [3,4,16]. In comparison with static pictures, videos showing chewing, swallowing, crispness, stretchiness, and juiciness offer a more abundant source of sensory information that allows the viewers to perceive the palatability and edibility more fully [13]. There has been evidence that seeing others eat is associated with positive perceptions about food and can impact purchasing behaviors [9,10]. This phenomenon might be particularly noticeable in the context of mukbang since this is essentially a form of media behavior aimed at observing someone else eating food [7,8], whereby viewers are presented not only with food cues but also eating pace, oral sound cues, and close-up sensations [5,17].

Prior mukbang research has mainly developed along three streams. The first line is on motivations to view mukbang, including company, fun, curiosity and voyeurism regarding overeating [7,9]. The second line explores mukbang’s effects on appetite, craving, hedonic hunger and overconsumption [6], and proposes that mukbang serves as an extreme food cue system capable of provoking appetitive responses and even binge eating behavior among its viewers [6,12]. A third line has started to focus on how to stylistically and sensorily vary food videos, such as changing the point of view, ASMR properties, and sensory-oriented presentations, which shows that these various presentation styles could affect motivational results and behavioral intentions [17,18]. Together, these studies provide an important foundation for understanding how mukbang affects consumers.

Nevertheless, several important gaps remain. First, prior research has largely treated mukbang as a broad and relatively homogeneous form of digital food content, paying limited attention to theoretically meaningful differences in presentation style [13,17,19]. In practice, however, mukbang videos vary in the extent to which they emphasize food-related sensory cues versus verbal, performative, and host-centered elements. To capture this variation, we distinguish between immersive mukbang and noisy mukbang as two different presentation logics. This distinction goes beyond existing categorizations by identifying a theoretically relevant difference in food-cue salience and sensory focus. Second, although prior studies have linked mukbang to appetite-, craving-, and impulse-related responses [6], less is known about the content-specific mechanism through which different mukbang types influence consumer behavior. We propose oral sensory arousal as a key mechanism because it more directly reflects the mouth-related sensory simulation triggered by mukbang content [20,21]. Third, existing research has focused more on the persuasive power of food cues themselves than on how their visual design shapes their effects [22,23]. In mukbang short videos, visual composition may determine whether attention is directed more toward the food or the host, thereby influencing the ways in which immersive mukbang is most effective.

To address these gaps, the present research draws on visual hunger theory to examine how mukbang type influences consumers’ immediate ordering intention, with oral sensory arousal as the mediator and visual composition as the moderator. We distinguish between immersive and noisy mukbang, proposing that immersive mukbang reduces verbal and performative distractions while emphasizing food-related details and oral sound cues, thereby heightening oral sensory arousal and increasing immediate ordering intention. We further argue that a food-centered (vs. human-centered) composition enhances the salience of food cues and strengthens this effect. These predictions are tested in three preregistered experiments using independent-samples *t*-tests, ANOVA, and PROCESS-based mediation and moderated mediation analyses [24]. Study 1 tests the main effect of mukbang type, Study 2 examines the mediating role of oral sensory arousal, and Study 3 investigates the moderating role of visual composition.

This research makes three main contributions. First, it extends visual hunger theory to the context of short-video food consumption by shifting attention from appetite-related responses to immediate ordering intention, a more behaviorally meaningful outcome in digital consumption settings. Second, it identifies oral sensory arousal as a content-specific mechanism through which mukbang type influences consumers’ ordering intention, thereby enriching the literature on digital food communication and multisensory consumer response. Third, it highlights visual composition as an important boundary condition, showing that the effectiveness of mukbang depends not only on presentation style but also on how visual attention is directed within the frame. Together, these contributions offer a more nuanced understanding of how short-video food content shapes consumer decision-making and provide useful implications for the design of digital food media [25,26].

## 2. Theoretical Background and Hypotheses

### 2.1. Visual Hunger Theory

Visual hunger theory suggests that exposure to food-related visual cues can trigger appetitive activation, reward anticipation, and eating readiness even in the absence of actual consumption [27]. Mere exposure to food may evoke a desire to eat, approach, or consume it. As established earlier studies have found out that images and videos of food rapidly attract attention and initiate psychological processes associated with reward and motivation, which makes food appear more attractive and alters behavior regarding intention to consume it [14,19,28].

The impact of visual hunger is also more pronounced in digital media environments. The content of social media, short-video applications and online food-ordering services reiterates stylized and intensified images of food, including extreme close-ups, saturated colors, texture presentations, and animated acts of eating [29,30]. Such stimuli boost expected pleasure and make people feel more prone to impulsive consumption behaviors [31]. So, visual hunger theory not only elucidates why food content triggers appetite but also clarifies how it might affect more behaviorally meaningful results like ordering intention [32,33].

In the present research, mukbang videos are treated as high-intensity digital food cues [6,7,8]. Unlike noisy mukbang, immersive mukbang is more likely to focus on food-related visual and audio cues more vividly, and is thus more likely to induce visual hunger [13,14]. This response may further manifest as oral sensory arousal, through which viewers become more aware of chewing, swallowing, and mouthfeel-related sensations [34,35], which in turn should enhance immediate ordering intention. At the same time, visual composition is what determines the salience of food cues. Compared with human-centered composition, food-centered composition makes food cues more salient and so is more likely to amplify the influence of immersive mukbang [36,37]. Additionally, the theory of visual hunger serves as the underlying theoretical framework that describes the relationship between mukbang type, oral sensory arousal, and immediate ordering intention in this paper.

### 2.2. Mukbang Type and Immediate Ordering Intention

In this research, we conceptualize mukbang type as a difference in presentation logic and distinguish between immersive mukbang and noisy mukbang. Immersive mukbang refers to a presentation style that emphasizes close-up food imagery, eating details, and oral sound cues while minimizing verbal and performative distractions [8,19]. By contrast, noisy mukbang refers to a presentation style that places greater emphasis on speech, expressive performance, and host-centered interaction, making food-related sensory cues comparatively less focal [5,6].

Based on visual hunger theory, food cues are more likely to trigger appetitive readiness and approach tendencies when they are vivid, salient, and relatively free from irrelevant distraction [21,27,34]. Immersive mukbang fits this condition because it concentrates viewers’ attention on close-up food imagery, eating details, and oral sound cues [17]. By doing so, it makes the sensory properties of the featured food more mentally accessible. This heightened focus on food-related cues should, in turn, increase viewers’ readiness to consume the food and strengthen their immediate ordering intention [38,39].

By contrast, although noisy mukbang may also attract viewers, a larger share of that attention is directed toward the host’s speech, expressive performance, and interaction rather than toward the food itself [12,13]. As a result, food-related sensory cues become comparatively less salient, weakening viewers’ mental simulation regarding consumption and reducing the likelihood of immediate ordering. Taken together, these differences suggest that immersive mukbang should be more effective than noisy mukbang in increasing consumers’ immediate ordering intention. Therefore, we propose the following hypothesis:

**H1.**  *Immersive (vs. noisy) mukbang is more likely to boost the immediate ordering intention.*

### 2.3. The Mediating Role of Oral Sensory Arousal

Oral sensory arousal is the subjective experience of mouth-related sensations (chewing, swallowing, crispness, softness, and juiciness) in consumers when they are exposed to food content. Even though these sensations are not induced by consumption itself, digital food media may create the same kind of oral sensations for the viewer with the help of visual and audio signals, as well as the feeling that they can almost perceive the texture and mouthfeel of the food [21,27,34,40,41]. In this regard, oral sensory arousal may be seen as an even more tangible extension of visual hunger.

We focus on oral sensory arousal rather than broader constructs such as general appetite or affect because this is more conceptually proximal to the defining characteristics of mukbang. While general appetite reflects an overall desire to eat and affect captures broad emotional reactions, oral sensory arousal specifically reflects the mouth-related sensory simulation triggered by chewing, swallowing, texture, and oral sound cues. Because these cues are central to mukbang content, oral sensory arousal provides a more precise explanation of how mukbang type translates into immediate ordering intention.

This mechanism is especially relevant in the context of mukbang because mukbang does not merely display food; it also presents the dynamic process of biting, chewing, and swallowing. These cues help viewers mentally simulate what the food would feel like in the mouth [13,16]. Immersive mukbang should be particularly effective in eliciting this response because it reduces verbal and performative distraction while giving greater prominence to food details and oral sound cues. This stronger sensory focus should lead viewers to experience greater oral sensory arousal than when viewing noisy mukbang [20,21].

Oral sensory arousal should also increase immediate ordering intention. In online food-consumption contexts, consumers cannot directly taste the product and therefore rely heavily on mental simulation to infer anticipated enjoyment and satisfaction. When a video evokes vivid mouth-related sensations such as chewing, swallowing, crispness, or juiciness, the food becomes easier to imagine consuming in the present moment [1,2,3]. This stronger sense of anticipated sensory experience should, in turn, increase the likelihood that viewers want to order the featured food immediately [10,14]. Therefore, we propose the following hypothesis:

**H2.**  *Oral sensory arousal mediates the effect of mukbang type on consumers’ immediate ordering intention.*

### 2.4. The Moderating Role of Visual Composition

Visual composition is the term used to describe the composition of the main visual focus in terms of its placement on a video frame [36,42]. Using the example of the mukbang short video, this paper differentiates between human-centered and food-centered compositions. Human-centered compositions emphasize the host’s face, actions, and expressive performance, whereas food-centered compositions place the food in a more visually dominant position. Existing studies have indicated that the manner in which the food is visually displayed exerts a significant impact on consumer perceptions, assessments, and purchase intentions. This is particularly prominent in digital ordering and social media environments, where appetitive and consumption-related trends are more susceptible to being triggered by food signals that are rendered more visually salient [36,42].

Visual composition should shape the effectiveness of mukbang type by influencing where viewers’ attention is directed [43,44]. When the video adopts a food-centered composition, the featured food occupies the dominant visual position, making its color, texture, and eating-related details more salient [45]. This stronger visual emphasis should amplify the sensory advantage of immersive mukbang and increase its ability to evoke oral sensory arousal and immediate ordering intention. By contrast, in a human-centered composition, more attention is directed to the host’s face, expression, and performance. This shifts attention away from the food itself and reduces the relative salience of food-related sensory cues. As a result, the positive effect of immersive mukbang should be weaker in a human-centered composition than in a food-centered composition. Therefore, visual composition is expected to moderate the effect of mukbang type on immediate ordering intention.

**H3.**  *Visual composition moderates the effect of mukbang video type on consumers’ immediate ordering intention; the positive effect of immersive mukbang emerges under food-centered composition but not under human-centered composition.*

**Figure 1:**
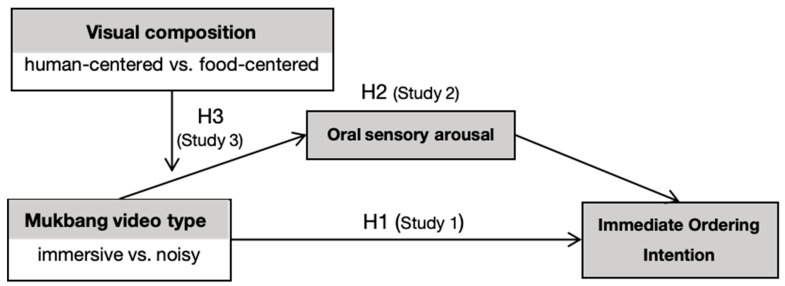
Research Model.

The research model is illustrated in Figure 1.

## 3. Overview of Studies

To improve readability and highlight the progressive logic of the empirical design, we provide an overview of the three studies before presenting the individual experiments. Study 1 tests the main effect of mukbang type on immediate ordering intention. Study 2 builds on this result by examining oral sensory arousal as a mediating mechanism. Study 3 further extends the model by introducing visual composition as a moderator and testing the overall moderated mediation framework. Table 1 summarizes the purpose, design, and main findings of the three studies.

## 4. Study 1: Main Effect of Mukbang Type on Immediate Ordering Intention

Study 1 aimed to examine the main effect of mukbang type on consumers’ immediate ordering intention. A one-factor between-subjects design was adopted where the mukbang type (immersive vs. noisy) was the independent variable.

### 4.1. Method

*Participants*. A priori power analysis using G*Power 3.1 (version 3.1.9.7.) indicated a minimum sample size of 128 for detecting a medium effect with 80% power [46]. We recruited 300 participants via Credamo [47] and retained 287 valid responses for the final analysis (*M_age_* = 31.7 years, SD = 7.48, 50.5% female), which exceeded the minimum requirement.

**Figure 2:**
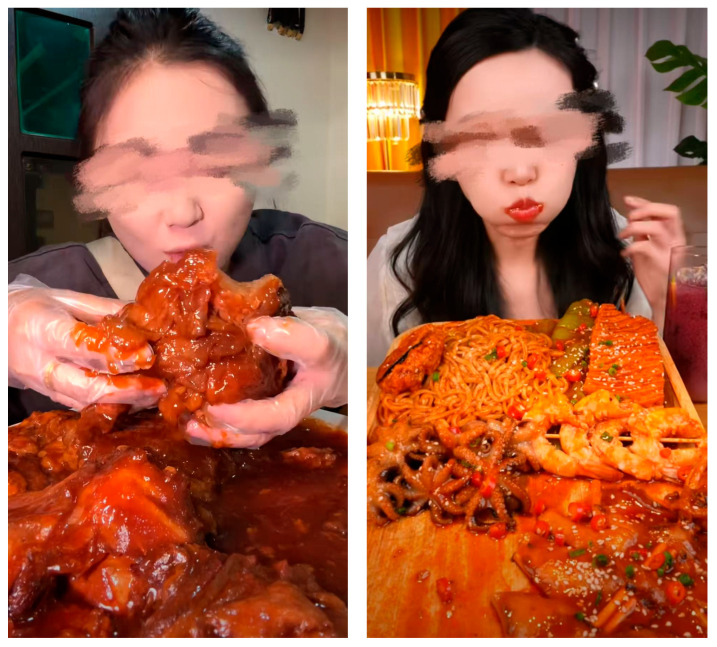
Stimulus materials for study 1 and study 2.

*Procedure.* Study 1 took place on Credamo, an experimental online study platform in China. Once the instructions were read and informed consent granted, participants were randomly allocated to one of the two conditions, i.e., immersive mukbang or noisy mukbang conditions. The stimuli consisted of two 40 s vertical short videos (1080 × 1920, MP4; see Figure 2). A 40 s duration was considered appropriate because this is consistent with the brief-exposure format of short-video platforms and provides sufficient time for viewers to process dynamic food cues. Prior research suggests that brief food-related video or motion-based presentations can influence visual attention, mental imagery, appetitive processing, and purchase intention [28,29,30,31]. The stimulus videos were edited with the assistance of a professional video editor and were matched as closely as possible in terms of food category, duration, resolution, and overall format. The intended difference was limited to presentation style, and the manipulation checks across studies further confirmed the effectiveness of this distinction. Specifically, the immersive mukbang video focused on close-up pictures of food, the consumption process and the sound of chewing, and the noisy mukbang video focused on verbal communication, emotional behavior, and performance interaction. The participants needed to watch the whole video before moving on. Subsequent to the viewing, they filled out the measures of immediate ordering intention, manipulation checks and general demographics. Before the data collection, the methodology, the hypotheses, the sample size, and the analysis plan in this study were registered in advance on the AsPredicted website (https://aspredicted.org/mb6gr2.pdf) (accessed on 26 March 2026) [24].

### 4.2. Measures

Immediate ordering intention was measured using a three-item, seven-point Likert scale (1 = strongly disagree; 7 = strongly agree): “I wish to order this food at this moment,” “I am ready to buy this food in the blink of an eye,” and “After seeing this video, I am determined to get this food” (α = 0.84) [6,47]. As a manipulation check, participants rated the overall style of the video on a seven-point scale (1 = noisy-oriented; 7 = immersive-oriented).

### 4.3. Results

*Manipulation Check*. An independent-samples *t*-test showed that participants in the immersive mukbang group perceived the video as significantly more immersive than those in the noisy mukbang group, indicating that the manipulation was successful. Specifically, participants in the immersive mukbang group (*M_immersive_* = 5.11, *SD* = 0.85) reported significantly higher perceived immersiveness than those in the noisy mukbang group (*M_noisy_* = 3.05, *SD* = 0.84, *t*(285) = −20.60, *p* < 0.001).

**Figure 3:**
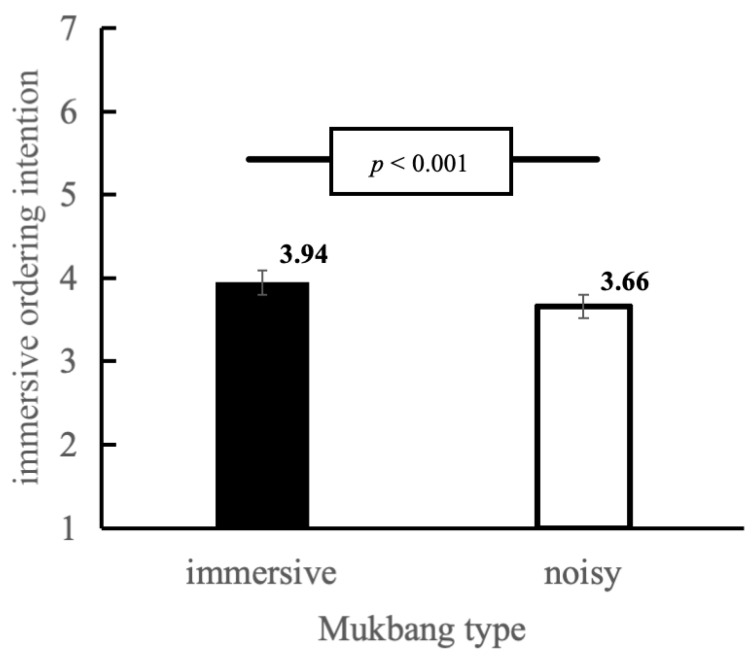
The effects of mukbang type on immediate ordering intention with standard error bars.

*Main Effects*. As shown in Figure 3, participants who viewed immersive mukbang reported a stronger intention to order the featured food immediately than those who viewed the noisy mukbang. Specifically, the immersive mukbang condition (*M_immersive_* = 3.94, *SD* = 0.48) elicited significantly greater immediate ordering intention than the noisy mukbang condition (*M_noisy_* = 3.66, *SD* = 0.52, *t*(285) = −4.57, *p* < 0.001). Thus, **H1** was supported.

### 4.4. Discussion

Study 1 provides initial evidence that immersive mukbang is more effective than noisy mukbang in increasing consumers’ immediate ordering intention. This finding offers preliminary support for the idea that not all mukbang content functions in the same way, and that differences in presentation style may shape immediate consumer responses. However, Study 1 only establishes the direct effect of mukbang type. To better understand why this effect occurs, Study 2 examines the mediating role of oral sensory arousal.

## 5. Study 2: Testing the Mediating Role of Oral Sensory Arousal

The objective of Study 2 was to evaluate how oral sensory arousal served as a mediator in the connection between mukbang type and immediate ordering intention. A one-factor between-subjects design was adopted where mukbang type (immersive vs. noisy) is an independent variable mediator of oral sensory arousal and immediate ordering intention is a dependent variable.

### 5.1. Method

*Participants*. A priori power analysis again indicated a minimum sample size of 128. To increase external validity, Study 2 was conducted offline at South China Agricultural University, Zhujiang College [48]. We recruited 150 participants and retained 142 valid responses for the final analysis (*M_age_* = 21.2 years, SD = 3.19, 52.1% female).

*Procedure*. Study 2 was conducted offline in a quiet room at South China Agricultural University, Zhujiang College. Participants were randomly assigned to the immersive or noisy mukbang conditions and viewed the same stimuli used in Study 1. After watching the video, they completed measures of oral sensory arousal and immediate ordering intention, as well as manipulation checks, and provided demographic information. Before conducting any data collection, the research design, hypotheses, sample size, and analysis plan were preregistered on the AsPredicted website (https://aspredicted.org/be9v5h.pdf) (accessed on 28 March 2026) [24].

### 5.2. Measures

The oral sensory arousal items were adapted from prior research on visual hunger and multisensory food perception [27,34] and reworded to fit the context of short-video exposure (1 = strongly disagree; 7 = strongly agree): “While watching the video, I could almost taste the food in my mouth,” “The video triggered my perception of chewing and swallowing very strongly,” and “Watching the video provided me with a vivid oral sensory experience” (α = 0.83). Immediate ordering intention was measured using the same scale as in Study 1 (α = 0.81). As a manipulation check, participants rated the overall style of the video on a seven-point scale (1 = noisy-oriented; 7 = immersive-oriented).

### 5.3. Results

*Manipulation Check*. Independent-samples *t*-test demonstrated that respondents in the immersive mukbang group (*M_immersive_* = 5.15, *SD* = 0.86) rated the video as significantly more immersive compared to those in the noisy mukbang group (*M_noisy_* = 3.04, *SD* = 0.82, *t*(140) = −15.03, *p* < 0.001). These results indicate that the manipulation was successful.

**Figure 4:**
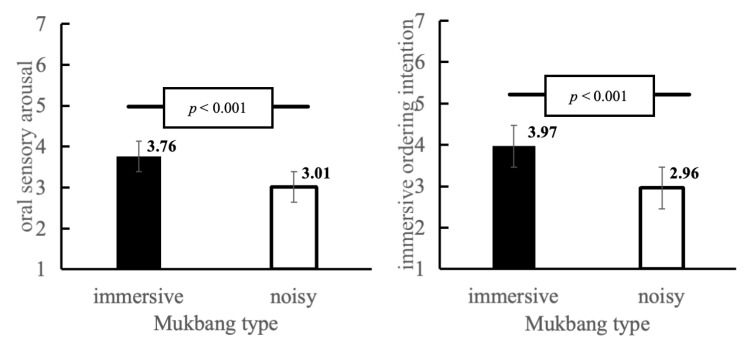
The effects of mukbang type on oral sensory arousal (**left**) and immediate ordering intention (**right**) with standard error bars.

*Main Effects*. Oral sensory arousal was used as the dependent variable in an independent-samples *t* test. The findings of Figure 4 indicate that those who viewed the immersive mukbang (*M_immersive_* = 3.76, *SD* = 0.95) had significantly greater oral sensory arousal compared to those who saw the noisy mukbang (*M_noisy_* = 3.01, *SD* = 0.91, *t*(140) = −4.80, *p* < 0.001). This result suggests that immersive mukbang elicits stronger oral sensory arousal than noisy mukbang. In the case of immediate ordering intention, it was found that those in the immersive mukbang group (*M_immersive_* = 3.97, *SD* = 0.51) reported significantly higher levels of immediate ordering intention than those in the noisy mukbang group (*M_noisy_* = 2.96, SD = 0.48, *t*(140) = −12.12, *p* < 0.001). This result further supports **H1**.

*Mediation Analysis*. The mediation analysis was carried out using PROCESS model 4 with 5000 bootstrap samples to examine **H2** [49]. The mediation analysis showed that oral sensory arousal helps explain why immersive mukbang increases immediate ordering intention. Specifically, mukbang type significantly predicted oral sensory arousal (*b* = 0.74, SE = 0.08, *p* < 0.001), and oral sensory arousal significantly predicted immediate ordering intention (*b* = 0.19, SE = 0.09, *p* < 0.05). Moreover, the indirect effect was significant (indirect effect = 0.16, 95% CI [0.012, 0.301]), supporting **H2**.

### 5.4. Discussion

Study 2 extends the findings of Study 1 by identifying oral sensory arousal as an underlying mechanism linking mukbang type to immediate ordering intention. Specifically, immersive mukbang increased immediate ordering intention not only directly, but also indirectly by eliciting stronger mouth-related sensory simulation. This finding suggests that the persuasive effect of mukbang is driven not simply by the general attractiveness of the food, but by the extent to which the video evokes a vivid oral sensory experience. Building on this result, Study 3 further examines whether visual composition serves as a boundary condition for this mechanism.

## 6. Study 3: Testing the Moderating Role of Visual Composition

The role of Study 3 is to evaluate the moderating effect of visual composition on the association between mukbang type and the immediate ordering intention. The design that was used was a two (mukbang type: immersive vs. noisy) by two (visual composition: food-centered vs. human-centered) between-subjects design. Mukbang type and visual composition acted as independent variables, the intermediary variable was oral sensory arousal, and the dependent variable was immediate ordering intention.

### 6.1. Method

*Participants*. A priori power analysis indicated that 128 participants would be sufficient for the factorial design [45]. Study 3 was conducted online via Credamo. We recruited 300 participants and retained 284 valid responses for the final analysis (*M_age_* = 28.9 years, *SD* = 8.49, 52.8% female).

**Figure 5:**
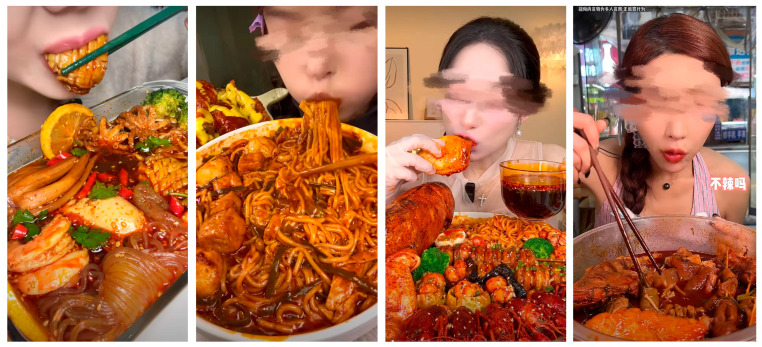
Stimulus materials for study 3. “视频内食物为多人食用 无浪费行为”: The food shown in the video was shared among multiple people, with no waste. “不辣吗”: Is it not spicy?

*Procedure*. Participants were randomly assigned to one of four conditions in a 2 (mukbang type: immersive vs. noisy) × 2 (visual composition: food-centered vs. human-centered) between-subjects design. The stimulus materials were four 40 s vertical short videos (1080 × 1920, MP4; see Figure 5). Mukbang type was manipulated in the same way as in the previous studies: the immersive condition emphasized close-up food imagery, eating details, and chewing sounds, whereas the noisy condition emphasized verbal expression, emotional display, and performance-oriented interaction. Visual composition was manipulated by varying whether the food or the host occupied the dominant visual position in the frame. After viewing the video, participants completed measures of oral sensory arousal and immediate ordering intention, as well as completing manipulation checks and providing demographic information. Responses that failed the attention check, showed abnormal completion times, or were duplicates were excluded. The study was preregistered on AsPredicted (https://aspredicted.org/vw936k.pdf) (date accessed on 28 March 2026) [24].

### 6.2. Measures

Immediate ordering intention was measured using the same scale as in Studies 1 and 2 (α = 0.87). Oral sensory arousal was measured using the same scale as in Study 2 (α = 0.91). Two single-item manipulation checks were also included. Participants rated the overall style of the video (1 = noisy-centered; 7 = immersive-centered) and the overall visual composition (1 = human-centered composition; 7 = food-centered composition).

### 6.3. Results

*Manipulation Checks*: The results of a set of independent-sample *t*-tests indicated that the immersive mukbang condition (*M_immersive_* = 5.08, *SD* = 0.82) was considered significantly more immersive compared to the noisy mukbang condition (*M_noisy_* = 2.97, *SD* = 0.83, *t*(282) = −21.53, *p* < 0.001). Also, participants in the food-centered composition group reported seeing the food as significantly more visually dominant (*M_food-centered_* = 5.05, *SD* = 0.82) than people in the human-centered composition group (*M_human-centered_* = 3.02, *SD* = 0.87, *t*(282) = −20.31, *p* < 0.001). These findings suggest that the two manipulations worked.

**Figure 6:**
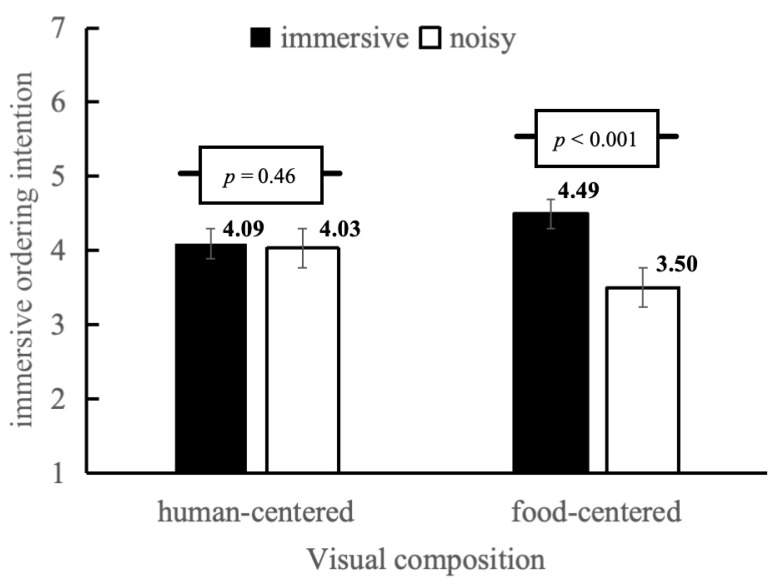
Interaction effects between mukbang type and visual composition on immediate ordering intention with standard error bars.

*Interaction Effects*. The effect of mukbang type on immediate ordering intention depended on visual composition. The next analysis was a two-way ANOVA where immediate ordering intention was the dependent variable. Figure 6 shows that the interaction effect of the two variables, namely mukbang type and visual composition, was statistically significant (F(1, 280) = 17.19, *p* < 0.001, *η_p_^2^* = 0.06). Simple-effect analyses also revealed that in cases where the visual composition was human-centered (W = 0), the difference in immediate ordering intention between immersive mukbang and noisy mukbang was not significant (*M_immersive_* = 4.09, *SD* = 0.51; *M_noisy_* = 4.03, *SD* = 0.49, *t*(136) = −0.74, *p* = 0.46). Conversely, if the visual composition was food-centered (W = 1), the immersion-mukbang and the noise-mukbang groups led to significantly different immediate ordering intentions (*M_immersive_* = 4.49 *SD* = 1.21 *M_noisy_* = 3.50 *SD* = 1.23, *t*(144) = −4.87, *p* < 0.001). Thus, H3 was supported.

*Moderated Mediation Analysis*. The moderated mediation analysis further showed that visual composition shaped the indirect effect of mukbang type through oral sensory arousal. In order to extend the conditionality of the indirect impact, another moderated mediation analysis was performed based on the PROCESS Model 7 with 5000 bootstrap samples [49]. It was found that the type of mukbang significantly predicted oral sensory arousal (*b* = 1.41, SE = 0.18, *p* < 0.001). However, it was found that the interaction between mukbang type and visual composition significantly predicted oral sensory arousal (*b* = 1.22, SE = 0.26, *p* < 0.001), and oral sensory arousal significantly predicted immediate ordering intention (*b* = 0.12, SE = 0.05, *p* < 0.05), which indicates that visual composition moderates the first-stage path between mukbang type and oral sensory arousal. Further analysis of the conditional indirect effect demonstrated that in cases with a human-centered visual composition (W = 0), the indirect effect of mukbang type on immediate ordering intention mediated through oral sensory arousal was insignificant (indirect effect = 0.19, 95% CI [−0.166, 0.546]). Conversely, in cases with a food-centered visual composition (W = 1), the indirect effect was found to be significant (indirect effect = 0.34, 95% CI [0.147, 0.773]). The index of moderated mediation was significant (index = 0.141, 95% CI [0.018, 0.253]). These findings provide further support for the moderated mediation structure outlined in this paper.

### 6.4. Discussion

Study 3 shows that the effect of mukbang type depends on its visual composition. Compared with a human-centered composition, a food-centered composition strengthens the positive effect of immersive mukbang on immediate ordering intention and supports the proposed moderated mediation pattern through oral sensory arousal. These findings suggest that the effectiveness of a mukbang depends not only on the content style, but also on how visual attention is directed within the frame. The broader theoretical implications of this boundary condition are discussed in the General Discussion.

## 7. General Discussion

### 7.1. Main Findings

Drawing on visual hunger theory, this research examined how mukbang type influences consumers’ immediate ordering intention and explored the mediating role of oral sensory arousal and the moderating role of visual composition. Across three preregistered studies, the findings consistently support the proposed framework.

First, Study 1 shows that immersive mukbang generates a stronger immediate ordering intention than noisy mukbang. This finding suggests that mukbang should not be treated as a homogeneous form of digital food content. Rather, immersive mukbang appears to be more effective because it makes food-related sensory cues more salient while reducing verbal and performative distractions, which is consistent with prior research on visual food cues and dynamic food presentation [13,14,16,38,39].

Second, Study 2 identifies oral sensory arousal as the mechanism underlying this effect. This result suggests that the influence of immersive mukbang is not driven merely by general liking or appetite, but by a more specific sensory process. By emphasizing chewing, swallowing, texture, and oral sound cues, immersive mukbang strengthens viewers’ mouth-related sensory simulation, which in turn increases immediate ordering intention [21,27,34,35].

Third, Study 3 shows that visual composition serves as an important boundary condition. The positive effect of immersive mukbang is stronger with a food-centered composition than with a human-centered composition, suggesting that the persuasive impact of mukbang depends not only on content style but also on how visual attention is directed within the frame. In this sense, visual composition functions as a meaningful condition shaping the salience and processing of digital food cues [22,23,36,45].

Taken together, these findings show that consumers’ immediate ordering intention in short-video food contexts is shaped by an interaction between content style, sensory activation, and visual design.

### 7.2. Theoretical Contributions

This research makes three main theoretical contributions. First, it extends visual hunger theory to the context of short-video-based digital food consumption. Prior research has primarily used visual hunger theory to explain appetite, craving, and readiness to eat in response to food cues. By contrast, the present study shifts attention to immediate ordering intention, a more behaviorally meaningful outcome in digital consumption settings. In doing so, it broadens the explanatory scope of visual hunger theory in consumer behavior research [13,14,16].

Second, this research identifies oral sensory arousal as a content-specific mediating mechanism. Compared with broader constructs such as appetite, liking, or perceived tastiness, oral sensory arousal more directly captures the mouth-related sensory activation triggered by chewing, swallowing, crispness, and oral sound cues in mukbang videos. By highlighting this mechanism, the study contributes to the literature on digital food communication and multisensory consumer response by clarifying how sensory-rich short-video content translates into immediate ordering intention [12,17,19,20].

Third, this research highlights visual composition as an important boundary condition of mukbang effectiveness. Although prior research has shown that visual food cues shape attention, evaluation, and consumption-related responses, less attention has been paid to how the distribution of visual focus between the host and the food influences these effects in short-video contexts. Our findings show that a food-centered composition strengthens the persuasive effect of immersive mukbang by increasing the salience of food-related sensory information. This suggests that visual design is not merely a presentational feature, but a theoretically meaningful factor that shapes how digital food cues are processed and how consumer intentions are formed [22,23,26].

### 7.3. Managerial Implications

This research offers several practical implications for food marketers, short-video creators, and online food platforms. Importantly, these implications are most directly relevant to content scenarios in which the goal is to stimulate immediate ordering intention rather than broader outcomes such as entertainment, follower growth, or influencer branding.

First, when the objective is short-term conversion, immersive presentation appears to be more effective than more performance-oriented or highly talkative food video formats. Videos that emphasize close-up food imagery, eating details, and oral sound cues may better translate viewer exposure into immediate consumption-oriented responses. This suggests that practitioners seeking to increase immediate ordering intention should reduce unnecessary verbal distraction and foreground sensory food information more clearly.

Second, visual composition deserves strategic attention. The findings show that immersive mukbang is especially effective when the food occupies the dominant position in the frame. For creators, restaurant brands, and food-delivery merchants using short-video content, this implies that a food-centered framing may be particularly useful in key moments of the video, such as thumbnails, opening shots, or sensory highlight scenes, where immediate viewer response is most important.

Third, the present findings should not be interpreted as suggesting that all food-related short videos should adopt the same format. In contexts where the goal is social engagement, follower growth, or influencer branding, more host-centered and expressive video styles may still be strategically effective. Our results instead suggest that immersive and food-centered presentation is especially useful in situations where immediate ordering or short-term conversion is the primary objective.

### 7.4. Limitations and Future Research

Despite the consistent findings across the three studies, several limitations should be acknowledged. First, the samples were collected in China, and one study relied on an offline university-based sample, which may limit the cross-cultural generalizability and external validity of the findings [47]. Future research could therefore test the proposed framework in different cultural settings and with more diverse participant groups. Second, this research focused on immediate ordering intention rather than actual purchasing behavior. Although this outcome is more behaviorally relevant than appetite or hunger, it does not fully capture real marketplace responses. Future studies could address this limitation by examining actual behavioral outcomes, such as click-through behavior, real-time ordering decisions, or conversion data [48]. Third, the present research focused on two mukbang styles and two types of visual composition, whereas real-world mukbang content may vary along many other dimensions, such as food type, host characteristics, ASMR intensity, platform context and perceived healthiness and tastiness [50]. Accordingly, future research could explore these additional content features and boundary conditions to develop a more comprehensive understanding of how short-video food content shapes consumer responses [17,18].

## Figures and Tables

**Table 1:** Overview of the three studies.

Study	Purpose	Design and Sample	Main Findings
Study 1	To test the main effect of mukbang type on immediate ordering intention	One-factor between-subjects design (immersive vs. noisy mukbang); *N* = 287	Immersive mukbang significantly increased immediate ordering intention compared with noisy mukbang.
Study 2	To examine the mediating role of oral sensory arousal	One-factor between-subjects design (immersive vs. noisy mukbang); *N* = 142	Oral sensory arousal mediated the positive effect of immersive mukbang on immediate ordering intention.
Study 3	To test the moderating role of visual composition and the overall moderated mediation	2 (mukbang type: immersive vs. noisy) × 2 (visual composition: food-centered vs. human-centered) between-subjects design; *N* = 284	The positive effect of immersive mukbang on immediate ordering intention was stronger under food-centered composition; the moderated mediation effect was supported.

## Data Availability

The original contributions presented in this study are included in the article. Further inquiries can be directed to the corresponding author.
